# B7 costimulation and intracellular indoleamine-2,3-dioxygenase (IDO) expression in peripheral blood of healthy pregnant and non-pregnant women

**DOI:** 10.1186/1471-2393-14-306

**Published:** 2014-09-04

**Authors:** Enikő Grozdics, László Berta, Anna Bajnok, Gábor Veres, István Ilisz, Péter Klivényi, János Rigó, László Vécsei, Tivadar Tulassay, Gergely Toldi

**Affiliations:** First Department of Pediatrics, Semmelweis University, Bókay u. 53-54, Budapest, H-1083 Hungary; Department of Neurology, Faculty of Medicine, Albert Szent-Györgyi Clinical Center, University of Szeged, Semmelweis u. 6, Szeged, H-6725 Hungary; Department of Inorganic and Analytical Chemistry, University of Szeged, Dóm tér 7, H-6720 Szeged, Hungary; First Department of Obstetrics and Gynecology, Semmelweis University, Baross u. 27, Budapest, H-1088 Hungary; MTA-SZTE Neuroscience Research Group, Semmelweis u. 6, Szeged, H-6725 Hungary; MTA-SE Pediatrics and Nephrology Research Group, Bókay u. 53-54, Budapest, H-1083 Hungary

**Keywords:** B7, CD28, CTLA-4, Indoleamine 2,3-dioxygenase, Kynurenine, Monocyte, T cell, Tryptophan

## Abstract

**Background:**

B7 costimulatory molecules are expressed on antigen presenting cells (APCs) and are important regulators of T cell activation. We investigated the role of the B7 family of costimulatory molecules in the development of the systemic maternal immune tolerance during healthy pregnancy (HP). We also aimed to investigate the intracellular expression of indoleamine-2,3-dioxygenase (IDO) and plasma levels of tryptophane (TRP), kynurenine (KYN) and kynurenic acid (KYNA), important molecules with immunoregulatory properties, in order to describe their potential contribution to the pregnancy-specific maternal immune tolerance.

**Methods:**

We determined the frequency of activated (CD11b+) monocytes expressing B7-1, B7-2, B7-H1, and B7-H2, and that of T cells and CD4+ T helper cells expressing CD28, CTLA-4, PD-1, and ICOS in peripheral blood samples of healthy pregnant (HP) and non-pregnant (NP) women using flow cytometry. We also examined the intracellular expression of IDO applying flow cytometry and plasma levels of TRP, KYN and KYNA using high-performance liquid chromatography.

**Results:**

A significant increase in the prevalence of CD28+ T cells was observed in HP compared to NP women. At the same time a decrease was shown in the expression of CTLA-4 on these cells. The frequency of CD80+ monocytes was lower in HP women. The prevalence of IDO-expressing T cells and monocytes was higher in HP compared to NP women. Plasma KYN, KYNA and TRP levels were lower, while at the same time, the KYN/TRP ratio was higher in HP than in NP women.

**Conclusions:**

Costimulation via CD28 may not contribute to the immunosuppressive environment, at least in the third trimester of pregnancy. The development of the pregnancy-specific immune tolerance in the mechanism of B7 costimulation may be more related to the altered expression of B7 proteins on APCs rather than that of their receptors on T cells. The elevated intracellular IDO expression in monocytes and T cells, as well as higher plasma enzymatic IDO activity are likely to contribute to the systemic immunosuppressive environment in the third trimester characteristic for healthy gestation.

**Electronic supplementary material:**

The online version of this article (doi:10.1186/1471-2393-14-306) contains supplementary material, which is available to authorized users.

## Background

Since the conceptus is half of foreign origins, presenting paternal antigens, it is considered a semi-allograft to maternal immunity. Therefore, a maternal immune tolerance must develop to avoid immunological rejection of the fetus. The alterations contributing to maternal tolerance are present not only at the maternal-fetal interface, but also at the systemic level. Several components of this pregnancy-specific immune tolerance have been described over the recent years [1]. One of the most important factors is the decreased level of activation of T cells compared to the non-pregnant state. The second and third trimesters of pregnancy are characterized by a shift of the inflammatory balance towards the anti-inflammatory direction via the upregulation of Th2 cells [2] and a decrease in the Th17/regulatory T cell (Treg) ratio [3]. The kinetics of calcium influx upon stimulation via the T cell receptor (TCR) is decreased in Th1 and CD8 cells compared to lymphocytes isolated from non-pregnant women [4]. Several other factors may account for the decreased level of peripheral T lymphocyte activation in healthy pregnancy (HP).

**Figure 1:**
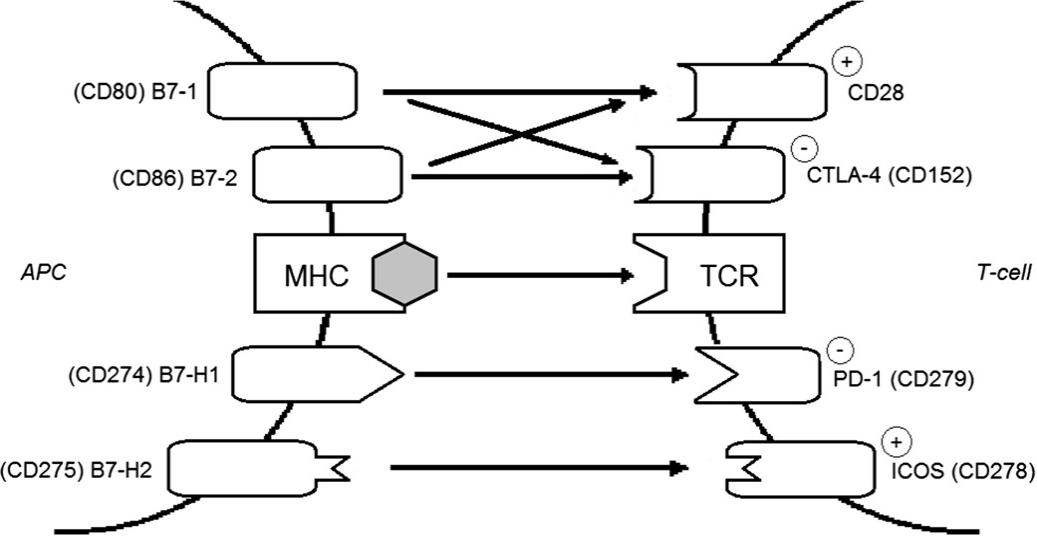
**B7 family proteins on antigen-presenting cells (APCs) and their cognate receptors on T cells.**

B7 costimulatory molecules are expressed on antigen presenting cells (APCs) and are important regulators of T cell activation (Figure 1). Besides the ligation of the T cell receptor (TCR) by the antigen associated with major histocompatibility complex (MHC) molecules, a costimulatory signal occurs through CD28, inducing the production of IL-2 in T cells, thus protecting them from apoptosis and anergy [5]. Without costimulation, the signal from the TCR induces the tolerance of T cells to their cognate antigen instead of being activated [6].

On the other hand, B7 proteins mediate not only stimulatory, but also inhibitory effects on T cells, thus potentially contributing to the lower reactivity of T lymphocytes in HP [5]. Upon the stimulation of the TCR, cytotoxic T lymphocyte antigen 4 (CTLA-4, CD154) becomes stabilized on the surface of T cells, thus competing with CD28 for B7 binding and blocking the costimulatory signal. The affinity of the inhibitory receptor, CTLA-4 is higher than that of CD28 for B7-1 (CD80) and B7-2 (CD86). Besides its competitive role, CTLA-4 also prevents T cell activation via the emission of direct inhibitory signals [7].

Another B7 family member, B7-H1 (CD274) possesses mostly inhibitory properties on T cells. This inhibitory function is mediated via programmed death-1 receptor (PD-1, CD279), which induces apoptosis or anergy of self-reactive T cells. B7-H2 (CD275) serves as the ligand for inducible costimulator of T cells (ICOS, CD278), and promotes T cell activation, differentiation, and effector responses [8]. In contrast to the costimulatory effect of CD28, ICOS most effectively induces IL-10 instead of IL-2 production [9].

Besides initiating signal transduction in T lymphocytes, B7-1 and B7-2 may back-signal into the APC and influence the local immune environment through induced expression of immunosuppressive factors independently of their effects on T cells [5]. Reverse signalling through B7-1 and B7-2 was shown to upregulate the tryptophan (TRP) catabolic enzyme, indoleamine 2,3-dioxygenase (IDO) [10]. The potent immunosuppressive activity of IDO was first identified in pregnancy, when it was demonstrated that inhibition of IDO abolished allogenic gestation in mice [11]. In the first steps of the kynurenine (KYN) pathway, TRP is transformed into KYN by IDO. KYN is then further metabolized by different enzymes. One of them is kynurenine aminotransferase, leading to the production of kynurenic acid (KYNA), a broad-spectrum endogenous antagonist of excitatory amino acid receptors [12, 13] with emerging recent implications in immunomodulation [14, 15]. The rate of TRP degradation, represented by the K/T (KYN to TRP) ratio, allows a good estimate of IDO activity. The local depletion of TRP and the production of pro-apoptotic TRP metabolites of the kynurenine pathway, such as 3-hydroxyanthranilic acid and quinolinic acid are among the mechanisms potentially responsible for the immunosuppressive effects related to IDO [14].

In this study, we aimed to determine the frequency of activated monocytes expressing B7-1, B7-2, B7-H1 and B-7H2 costimulatory molecules, as well as that of T cells and T helper cells expressing CD28, CTLA-4, PD-1 and ICOS in peripheral blood samples of HP compared to non-pregnant (NP) women. We also investigated the intracellular expression of IDO in activated monocytes and T cells, as well as plasma levels of TRP, KYN and KYNA.

## Methods

### Sample collection

Peripheral blood samples were taken from 20 healthy pregnant (HP) women in the third trimester and 14 age-matched, healthy non-pregnant (NP) women between 01/2012 and 08/2012. The latter group was synchronized in terms of menstrual cycle for the luteal phase. Clinical characteristics of participants are summarized in Table 1. Informed consent was obtained from all subjects, and our study was reviewed and approved by an independent ethical committee of the institution (Scientific and Research Ethics Committee, Semmelweis University, Budapest, Hungary). The study was adhered to the tenets of the most recent revision of the Declaration of Helsinki and to the STROBE guidelines for observational studies (checklist included as Additional file 1).

**Table 1:** **Clinical characteristics of non-pregnant and healthy pregnant women**

	Non-pregnant women (n = 14)	Healthy pregnant women (n = 20)
Age (years)	32 (27–34)	33.5 (30–36)
No. of primiparas	-	12 (60%)
Gestational age at blood collection (weeks)	-	36 (34–37)
Gestational age at delivery (weeks)	-	39 (38–40)
Fetal birth weight (grams)	-	3180 (2915–3725)

Data are presented as median (interquartile range) for continuous variables and as number (percentage) for categorical variables.

### PBMC isolation

Peripheral blood mononuclear cells (PBMCs) were separated by a standard density gradient centrifugation (Ficoll Paque, Amersham Biosciences AB, Uppsala, Sweden, 25 minutes, 400 *g*, 22°C) from freshly drawn blood collected in lithium heparin-treated tubes (BD Vacutainer, BD Biosciences, San Jose, CA, USA). Cells were kept at −80°C in Fetal Bovine Serum containing 10% DMSO until analysis. After thawing, cells were washed twice in phosphate-buffered saline and their viability was assessed by trypan blue exclusion (consistently > 90%).

### Flow cytometry

**Figure 2:**
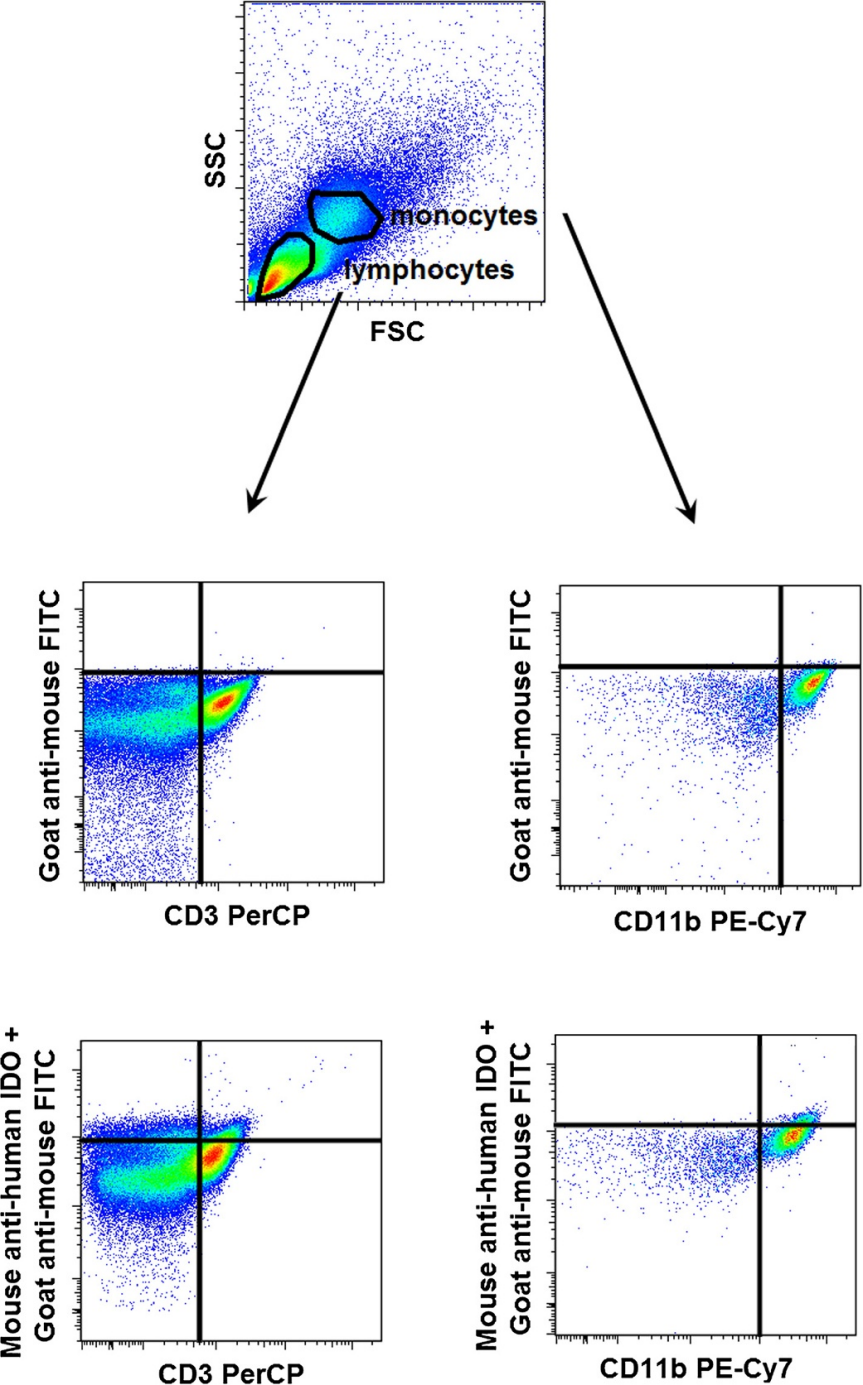
**Indoleamine-2,3-dioxygenase (IDO) enzyme expression in CD3+ lymphocytes and CD11b + monocytes measured by flow cytometry.** FSC – forward scatter characteristics, SSC – side scatter characteristics.

PBMCs were stained for 30 min at room temperature in the dark with PerCP-conjugated CD3, PE Cy7-conjugated CD4, PE-conjugated CD28, APC-conjugated CD152 (CTLA-4), FITC-conjugated CD278 (ICOS) and APC-Cy7-conjugated CD279 (PD-1) mAbs, or PerCP-conjugated CD3, PE Cy7-conjugated CD11b, APC-conjugated CD80 (B7-1) and PE-conjugated CD275 (B7-H2) mAbs, or PerCP-conjugated CD3, PE Cy7-conjugated CD11b, APC-conjugated CD86 (B7-2) and PE-conjugated CD274 (B7-H1) mAbs in separate tubes, respectively (BioLegend, San Diego, CA, USA). After washing, cells were fixed with Fixation/Permeabilization solution and treated with Permeabilization Buffer according to the manufacturer’s instructions (eBioscience, San Diego, CA, USA). They were then stained with a mouse anti-human IDO monoclonal antibody (Millipore, USA) for 30 min at 4°C in the dark. After washing, cells were stained with FITC-labelled goat anti-mouse antibody (Millipore, USA) for 15 min at 4°C in the dark. After washing, cells were analyzed on a BD FACSAria flow cytometer (BD Biosciences) equipped with 488 nm and 633 nm excitation lasers. Data were processed using the FACSDiVa software. 100000 cells were recorded. The populations of lymphocytes and monocytes were gated from PBMCs according to Forward Scatter Characteristics and Side Scatter Characteristics. As control of FITC-labelled goat anti-mouse specificity staining, PBMCs were incubated with surface antibodies and FITC-labelled goat anti-mouse antibody in the absence of mouse anti-human IDO monoclonal antibody. Figure 2 represents the measurement of intracellular IDO expression by flow cytometry.

### High-performance liquid chromatography (HPLC)

The investigated reference compounds (L-TRP, L-KYN sulfate salt, KYNA) and zinc acetate dihydrate were purchased from Sigma-Aldrich (Saint Louis, MO, USA), acetonitrile and perchloric acid (PCA) were purchased from Scharlau (Barcelona, Spain) and acetic acid was purchased from VWR International (Radnar, PA, USA).

Plasma samples were stored at −80°C until analysis. Before analysis, the samples were thawed and after a brief vortex 300 μl of plasma sample was ‘shot’ onto 700 μl precipitation solvent (containing 3.57 w/w% PCA and 2.857 μM 3-nitro-L-tyrosine as internal standard). Following that the samples were centrifuged at 13000 *g* for 10 min at 4°C, and the supernatant was collected.

**Figure 3:**
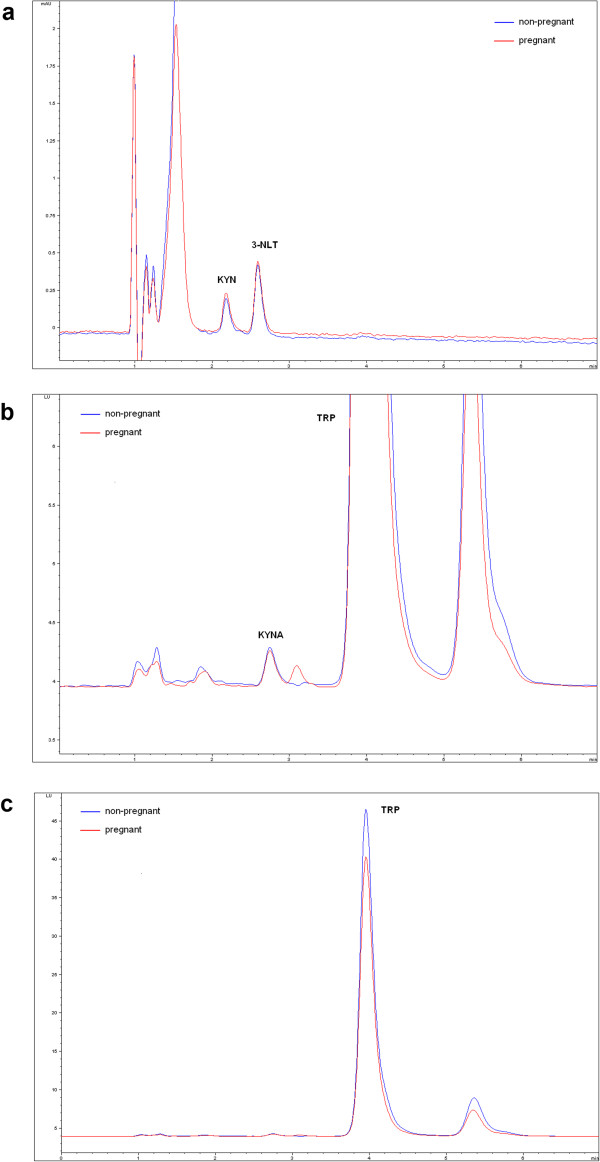
**Chromatograms of the investigated molecules. a**. represents the chromatogram of kynurenine (KYN) and the internal standard 3-nitro-L-tyrosine (3-NLT), made by UV detector (x: time (min), y: miliAbsorbanceUnit (mAU)). **b** and **c**. shows the chromatogram of kynurenic acid (KYNA) and tryptophan (TRP), respectively, made by fluorescent detector (x: time (min), y: Luminescence Unit (LU)).

The KYN, KYNA and TRP concentrations of the samples were quantified based on the slightly modified method of Herve et al. [16], with an Agilent 1100 HPLC system (Agilent Technologies, Santa Clara, CA, USA). The system was equipped with a fluorescent and a UV detector, the former was applied for the determination of KYNA and TRP, and the latter for the determination of KYN and the internal standard. Chromatographic separations were performed on an Onyx Monolithic C18 column, 100 mm × 4.6 mm I.D. (Phenomenex Inc., Torrance, CA, USA) after passage through a Hypersil ODS pre-column, 20 mm × 2.1 mm I.D., 5 μm particle size (Agilent Technologies, Santa Clara, CA, USA) with a mobile phase composition of 0.2 M zinc acetate/ACN = 95/5 (v/v%) with a pH adjusted to 6.2 with glacial acetic acid, applying isocratic elution. The flow rate and the injection volume were 1.5 ml/min and 20 μl, respectively. The fluorescent detector was set at excitation and emission wavelengths of 344 nm and 398 nm, and after 3.5 min of each run the wavelengths were changed to 254 nm and 398 nm. The UV detector was set at a wavelength of 365 nm. Figure 3 shows representative chromatograms of the measured metabolites.

### HPLC method validation

#### Calibration curve and linearity

Calibrants were prepared at 6 different concentration levels, from 1 to 100 nM, 0.1 to 5 μM, 5 to 50 μM and 0.5 to 7.5 μM for KYNA, KYN, TRP and the internal standard, respectively. Three parallel injections of each solution were made under the chromatographic conditions described above. The peak area responses were plotted against the corresponding concentration, and the linear regression computations were carried out by the least square method with the R software [17]. Very good linearity was observed throughout the investigated concentration ranges for KYN, KYNA, TRP and the internal standard when either fluorescence or UV detection was applied.

#### Selectivity

The selectivity of the method was checked by comparing the chromatograms of KYN, KYNA, TRP and the internal standard for a blank plasma sample and those for a spiked plasma sample. All compounds could be detected in their own selected chromatograms without any significant interference.

#### LOD and LLOQ

Limit of detection (LOD) and lower limit of quantitation (LLOQ) was determined via signal-to-noise ratio with threshold 3 and 10, according to the ICH guidelines [18]. The LOD was 100, 1 and 15 nM, while LLOQ was 275, 3.75 and 35 nM for KYN, KYNA and TRP, respectively.

#### Precision

Replicate HPLC analysis showed that the relative standard deviation was ≤ 2.2% for the peak area response and ≤ 0.1% for the retention time.

#### Recovery

The relative recoveries were estimated by measuring spiked samples of KYN, KYNA and TRP at 2 different concentrations with 3 replicates of each. No significant differences were observed for the lower and higher concentrations. The recoveries ranged from 108 to 110%, 86 to 91% and 85 to 89% for KYN, KYNA and TRP, respectively.

### Statistics

Data are expressed as median and interquartile range. The sample size was estimated to achieve 80% power with 0.45 effect size to detect differences between sample populations. Comparisons between sample populations were made with Mann–Whitney tests. Correlation analyses were performed using Spearman tests. p-values less than 0.05 were considered significant. Statistics were calculated using the STATISTICA software (version 8.0; StatSoft, Inc., Tulsa, Oklahoma, USA).

## Results

**Figure 4:**
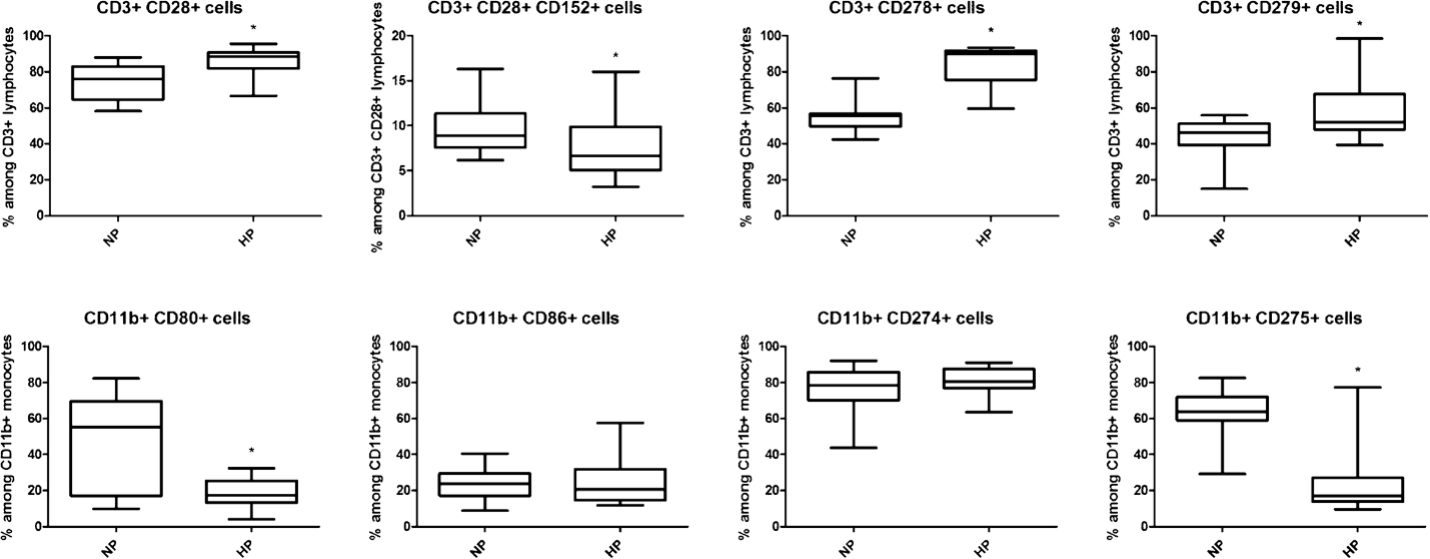
**Frequency of the investigated cell surface markers on CD3+ lymphocytes and CD11b + monocytes.** Horizontal line – median, box – interquartile range, whisker – range. NP – non-pregnant women, HP – healthy pregnant women. *p < 0.05 vs NP.

Our results are detailed in Tables 2 and 3 and Figure 4. A significant increase in the prevalence of CD28+ T cells was observed in HP compared to NP women. At the same time a decrease was shown in the expression of CD152 on these cells. The prevalence of both CD278+ and CD279+ T cells was higher in HP than in NP women.

**Table 2:** **Frequency of the investigated cell surface and intracellular markers**

	Non-pregnant women (n = 14)	Healthy pregnant women (n = 20)
CD3+ CD28+ cells/CD3+ lymphocytes	76.0 (64.7-82.9)%	88.4* (81.8-90.6)%
CD3+ CD28+ CD152+ cells/CD3+ CD28+ lymphocytes	8.90 (7.57-11.4)%	6.64* (5.07-9.89)%
CD3+ CD278+ cells/CD3+ lymphocytes	55.7 (49.5-56.7)%	89.9* (75.7-91.6)%
CD3+ CD279+ cells/CD3+ lymphocytes	46.3 (39.3-51.2)%	51.9* (47.9-67.7)%
CD4+ CD28+ cells/CD4+ lymphocytes	97.8 (96.7-98.8)%	97.3 (93.0-99.2)%
CD4+ CD28+ CD152+ cells/CD4+ CD28+ lymphocytes	6.92 (4.93-8.63)%	5.61 (3.65-9.09)%
CD4+ CD278+ cells/CD4+ lymphocytes	56.7 (52.8-59.5)%	87.7* (76.3-92.2)%
CD4+ CD279+ cells/CD4+ lymphocytes	49.4 (42.1-54.5)%	44.2 (37.5-65.2)%
CD11b + CD80+ cells/CD11b + monocytes	55.5 (17.3-69.8)%	17.6* (13.6-25.4)%
CD11b + CD86+ cells/CD11b + monocytes	23.8 (17.3-29.5)%	20.7 (14.9-31.9)%
CD11b + CD274+ cells/CD11b + monocytes	78.4 (70.3-85.9)%	80.7 (77.2-87.8)%
CD11b + CD275+ cells/CD11b + monocytes	63.8 (59.0-72.1)%	17.3* (14.1-27.0)%
CD3+ IDO + cells/CD3+ lymphocytes	5.90 (2.65-16.9)%	24.1* (13.3-56.4)%
IDO mean fluorescence intensity in CD3+ IDO + cells (arbitrary unit)	9888 (7482–11475)	62500* (19800–84475)
CD11b + IDO + cells/CD11b + monocytes	2.59 (1.99-11.7)%	22.5* (13.6-50.5)%
IDO mean fluorescence intensity in CD11b + IDO + cells (arbitrary unit)	21400 (19075–23875)	70450* (38775–114000)

*p < 0.05 versus non-pregnant women. Data are presented as median (interquartile range). IDO – indoleamine-2,3-dioxygenase, MFI – mean fluorescence intensity.

**Table 3:** **Plasma levels of kynurenine (KYN), kynurenic acid (KYNA) and tryptophan (TRP)**

	Non-pregnant women (n = 14)	Healthy pregnant women (n = 20)
KYN (uM)	1.80 (1.70-2.08)	1.55* (1.31-1.83)
KYNA (nM)	29.8 (25.6-47.7)	18.8* (15.0-23.0)
TRP (uM)	54.1 (49.0-59.3)	38.6* (33.9-42.5)
K/T ratio	0.037 (0.032-0.040)	0.041* (0.038-0.045)

*p < 0.05 versus non-pregnant women. Data are presented as median (interquartile range).

Within the CD4 subset, the ratio of CD28+, CD28+ CD152+ and CD279+ cells was comparable in HP and NP women, while that of CD278+ cells was higher in HP than in NP individuals.

The frequency of both CD80+ and CD275+ monocytes was lower in HP women, however, no difference was observed regarding CD86+ and CD274+ monocytes.

The prevalence of IDO-expressing T cells and monocytes was higher in HP compared to NP women. At the same time, the mean fluorescence intensity (MFI) values for IDO were also significantly higher in both cell subsets in HP.

Plasma KYN, KYNA and TRP levels were lower, while at the same time, the K/T ratio was higher in HP than in NP women.

In order to explore whether reverse signalling via CD80 and CD86 is present in monocytes, correlation analyses were performed. However, we could not detect a correlation between the frequency of CD80+ or CD86+ monocytes and the frequency of IDO-expressing T cells or monocytes or the MFI of IDO in the investigated study groups.

## Discussion

In this study, we aimed to characterize the prevalence of B7 costimulatory molecules on monocytes and their corresponding receptors on T lymphocytes in HP compared to NP women, as well as the intracellular expression of IDO and plasma levels of TRP, KYN and KYNA, important molecules with immunoregulatory properties, in order to describe their potential contribution to the pregnancy-specific maternal immune tolerance. Pregnancy is an immunosuppressive state, with well known alterations in the prevalence and function of T lymphocytes [1]. We assumed that alterations in costimulation mechanisms via B7 proteins might contribute to the lower level of T lymphocyte activation compared to NP women.

However, surprisingly, the expression of CD28 was increased, while that of CTLA-4 was decreased on T lymphocytes isolated from HP women. This finding indicates that costimulation via CD28 is of great importance also during pregnancy in T cells, and it may not contribute to the immunosuppressive environment characteristic for gestation. At the same time, the expression of B7-1 was decreased on HP monocytes, while that of B7-2 was unaltered, which might reduce the intensity of costimulation via CD28.

In cases of miscarriage, the expression of B7-2 was found to be highly upregulated at the fetomaternal interface and this was associated with high levels of Th1 cytokines (IL-2 and IFN-gamma) and low levels of Th2 cytokines (IL-4 and IL-10) [19]. Furthermore, it was reported that in vivo blockade of B7-2 costimulation shifted the cytokine balance from a Th1 to a Th2 predominance at the fetomaternal interface, and expanded peripheral CD4+ CD25+ regulatory T cells. Thus, reduction in the level or function of B7-2 appears to be advantageous to HP in the first half of pregnancy. We could not, however, demonstrate this reduction in third trimester peripheral blood HP samples.

The expression of ICOS, a stimulator of T cell activation was strongly elevated in HP, while that of its corresponding costimulatory molecule, B7-H2 was strongly decreased on HP monocytes. Since ICOS most effectively induces IL-10 instead of IL-2 production [9], its higher level may contribute to the Th2 shift observed in the third trimester of HP [1]. The frequency of PD-1 expressing T lymphocytes was also elevated in HP. The inhibitory effect of this receptor may play a role in inhibiting the activation of T cells during gestation. Interestingly, Taglauer et al. demonstrated that the expression of PD-1 expression on CD3 cells was low in non-pregnant endometrium but increased in first-trimester decidua and remained elevated in term decidua. Additionally, higher relative proportions of term decidual CD8bright, CD4, and Treg cells expressed PD-1 in comparison to autologous peripheral blood, further strengthening the role of this molecule in the development of maternal immune tolerance [20].

The prevalence of IDO-producing T cells and monocytes was elevated in HP compared to NP samples. The well-known immunosuppressive activity of this enzyme may play an important role in the development of pregnancy-specific immune tolerance towards the developing fetus. IDO is a key enzyme in the catabolism of tryptophan and initiates the production of kynurenines. These metabolites have several immunological and non-immunological regulatory functions. By locally depleting TRP and increasing the levels of KYN and its metabolites, IDO provides a suppression of T cell-mediated immune response via inhibiting the proliferation and inducing the apoptosis of activated T cells, as well as promoting the development of regulatory T cells and tolerogenic DCs [21]. Furthermore, we found that not only the prevalence of IDO-producing cells, but also the intracellular amount of IDO is elevated in HP (represented by the higher MFI values compared to NP women).

KYN, KYNA, and especially TRP levels were decreased in HP, resulting in an elevated K/T ratio compared to NP. As noted above, the depletion of TRP may directly contribute to the immunosuppressive environment in HP. The increase in IDO activity in HP (represented by the elevated K/T ratio) corresponds well with our finding of higher IDO-expressing T cell and monocyte numbers in HP. Interestingly, although the prevalence of IDO-producing T cells was decreased in our earlier study in PE compared to HP [22], KYN, KYNA and TRP levels, as well as the K/T ratio were comparable in HP and PE (1.55 (1.31-1.83) vs. 1.67 (1.33-1.93) uM, 18.8 (15.0-23.0) vs. 22.3 (16.7-28.5) nM, 38.6 (33.9-42.5) vs. 37.1 (30.0-41.3) uM, 0.041 (0.038-0.045) vs. 0.044 (0.036-0.049), respectively). Hence, the alterations observed in the frequency of IDO-producing cells are not reflected by the enzymatic activity of IDO in PE, in contrast to HP.

Another reason for decreased KYN, KYNA and TRP levels in HP compared to NP may be the fact that these molecules cross the placenta via yet partly unidentified mechanisms and transporters, and contribute to the development of the decreased immune responsiveness of the fetus. Our unpublished recent data, indicating elevated KYN, KYNA and TRP levels in cord blood of healthy term neonates compared to adult peripheral blood may support this assumption. However, further experimental data are needed to confirm or refute this hypothesis.

The engagement of B7-1 and B7-2 by CTLA-4 induces back signalling into the monocyte and promotes the production of IFN-gamma, which acts in an autocrine or paracrine manner to upregulate IDO expression, thereby initiating the degradation of TRP and resulting in an immunosuppressive effect as described above. Thus, the interaction between CTLA-4 and B7 proteins plays two different roles: first, at the level of T cells where CTLA-4 as a negative receptor regulates TCR signal transduction; second, at the level of APCs where CTLA-4 as a ligand signals to the APC to induce IDO expression [23]. We hypothesized that reverse signalling may play a role in the higher IDO activity and expression observed in HP. However, correlation analyses with B7-1 and B7-2 expression did not support this notion.

## Conclusions

In conclusion, costimulation via CD28 may not contribute to the immunosuppressive environment, at least in the last stage of pregnancy. Based on our findings, the development of the pregnancy-specific immune tolerance in the mechanism of B7 costimulation may be more related to the altered expression of B7 proteins on APCs rather than that of their receptors on T cells. The elevated intracellular IDO expression in monocytes and T cells, as well as higher plasma enzymatic IDO activity are likely to contribute to the systemic immunosuppressive environment in the third trimester characteristic for healthy gestation.

## Electronic supplementary material

Additional file 1: **STROBE Statement—checklist of items that should be included in reports of observational studies.** (DOC 98 KB)

## Abbreviations

APC: Antigen presenting cell
CTLA-4: Cytotoxic T lymphocyte antigen 4
HP: Healthy pregnancy
HPLC: High-performance liquid chromatography
ICOS: Inducible costimulator of T cells
IDO: Indoleamine-2,3-dioxygenase
KYN: Kynurenine
KYNA: Kynurenic acid
LOD: Limit of detection
LLOQ: Lower limit of quantitation
MFI: Mean fluorescence intensity
MHC: Major histocompatibility complex
NP: Non-pregnant
PBMC: Peripheral blood mononuclear cell
PCA: Perchloric acid
PD-1: Programmed death-1 receptor
TCR: T cell receptor
Treg: Regulatory T cell
TRP: Tryptophane.
